# Adaptation of the Information, Motivation, and Behavioral Skills Framework for Understanding COVID-19 Prevention Behaviors among Youth and Young Adults by Sexual Identity, Gender Identity and Race/Ethnicity

**DOI:** 10.3390/sexes4040042

**Published:** 2023-12-12

**Authors:** Gregory Phillips, Jiayi Xu, Michael G. Curtis, Megan M. Ruprecht, Shahin Davoudpour, Joseph Choi, Kay Quiballo, Sophia Huang, Kathryn Macapagal

**Affiliations:** Department of Medical Social Sciences, Northwestern University, Evanston, IL 60208, USA

**Keywords:** Information–Motivation–Behavioral Skills (IMB) model, IMB model, COVID-19, COVID-19 prevention, prevention intervention, youth, adolescents, prevention behaviors

## Abstract

Youth and young adults (YYA) have been uniquely affected by COVID-19. Behavioral models have not yet been applied to understand YYA’s preventive behavior, though the Information–Motivation–Behavior (IMB) model may be appropriate. We used data from a national, diverse survey of COVID-19 effects and prevention behaviors in YYA ages 14–24 (*n* = 1026) and conducted an exploratory factor analysis and bivariate linear regressions to assess the association between demographics and IMB measures. Significant differences by sexual identity were identified, with bisexual/pansexual, gay/lesbian, and queer populations reporting significantly lower prevention stigma, in comparison to their straight respondents. Non-binary respondents (vs. women/girls) and transgender and gender diverse individuals (vs. cisgender) had significantly higher intentions to social distance. Racial/ethnicity differences were also found in lower prevention efficacy beliefs (Latinx and multiracial vs. white), and lower motivation norms (Black and Latinx vs. white). Our findings about critical disparities in IMB measures provide recommendations for future prevention research, practice, and policy development in response to the pandemic, particularly related to highly marginalized communities.

## 1. Introduction

Since being declared a pandemic in March 2020, SARS-CoV-2 (COVID-19) has claimed over 1 million lives in the US and has severely impacted countless others, [1] causing widespread financial, emotional, and social turmoil [2,3]. However, not all populations have been equally affected. Youth and young adults (YYA) ages 14–24 years may not experience the highest rates of COVID-19-related morbidity or mortality, but they face a multitude of other adverse impacts due to the disruptions caused by the pandemic [4]. YYA may be at risk of developing multisystem inflammatory syndrome in children (MIS-C) [5] or “Long COVID” [6]. One report suggests that 1 in 5 previously healthy young adults experienced prolonged symptoms following COVID-19 infection [7]. YYA represent a critical population in which to better understand and support COVID-19 prevention. This is especially critical within minoritized communities, such as sexual and gender minority (SGM), Black, Hispanic/Latinx, and Native American populations, who are at greater risk for COVID-19-related harms such as decreased access to medical services and difficulty accessing food and supplies [8-11].

COVID-19 can be prevented by avoiding crowds and gatherings, using masks and physical distancing, self-isolating and quarantining, frequent testing, and keeping up to date with the proper vaccination guidelines [12]. Despite the high efficacy of these behaviors and the elevated risk to this age group, YYA may be less likely to engage in recommended mitigation behaviors, such as social distancing, mask wearing, and vaccination [13,14]. Given their developmental stage, which is marked by increases in independence while still developing impulse control, YYA may have difficulty internalizing and acting upon risk-reduction messaging [15]. Engagement in COVID-19 protective behaviors may be complicated by early COVID-19 messaging, indicating that YYA were not at a high risk for illness [16]. Of note, disparities in preventive behaviors among YYA may suggest the importance of looking at differences in mitigation behaviors by demographic groups. Potentially due to structural differences in access, studies have found that racial/ethnic minority populations were less likely to know where to obtain COVID-19 testing, less likely to engage in self-isolation when sick [17], and less likely to have appropriate sanitation supplies [11,17], compared to white populations. Some research suggests that SGM populations have less access to healthcare providers, potentially indicating lower prevention support [11]. Differences in testing and vaccination may also exist by sexual orientation and gender identity [10]. This underscores the importance of understanding barriers to prevention and mitigation which may exist for different vulnerable populations.

One dominant approach in understanding health promotion behavior is the use of the Information–Motivation–Behavioral Skills (IMB) model, an integrative model originally created for understanding HIV risk and prevention behaviors [18]. IMB posits that the mechanism behind risk-taking behavior change is a function of three factors: (1) Information, or how much someone knows about a health risk and its prevention behavior, (2) motivation to engage in prevention behavior, including attitudes and subjective norms, and (3) behavioral skills, including self-efficacy to engage in the desired behavior [19]. In practice, IMB suggests that positive and negative perception of health behaviors and interventions has a direct effect on the health outcomes of participants [20]. The IMB framework has effectively informed strategies to reduce HIV risk behaviors and to increase preventive measures, such as condom use within an array of diverse populations, and has been highly effective in YYA populations in particular [21,22].

Although IMB was initially developed to study HIV-risk related behavior change and health promotion, it has since been adapted to study the prevention of many other infectious and chronic diseases in YYA [23], including diabetes [24], human papillomavirus (HPV) [25], and mental illness [26]. Some studies have also began to use the IMB model to understand COVID-19 risk and prevention behaviors [27,28]; this includes the use of personal protective equipment (PPE), like masks [29], adherence to social distancing [20], and vaccine hesitancy [30]. Preliminary work suggests that the IMB model could aid in understanding and promoting prevention behaviors for COVID-19 among YYA [20,31]. However, while the IMB model has been used extensively in youth populations [21,22], it has not been applied to this group in the context of COVID-19 risk and prevention behaviors. The evidence shows compliance with COVID-19 mitigation strategies is less than optimal in the US [32]. In response, we aimed to (1) develop a measure to assess COVID-19 information, motivation, and behavioral skills, and (2) explore the role of IMB factors in COVID-19 mitigation strategies for a diverse population of YYA ages 14–24. This addition to the literature will help us identify key places for intervention, which are vital to minimizing the harmful impact of the pandemic and providing the groundwork for future interventions for different youth populations.

## 2. Materials and Methods

### 2.1. Participants and Procedure

Baseline data for the Youth and Young Adults COVID-19 Study were collected between 2/2021 and 3/2022 using Research Electronic Data Capture (REDCap). Eligibility criteria were being 14 to 24 years of age, residing in the United States/US territories, having access to the internet, being willing to complete a follow-up survey in 6 months, and providing informed consent. Participants were recruited through paid social media advertisements, outreach with organizations that served LGBTQ+, Indigenous, and Latinx youth, and an existing participant registry maintained by the study team. Advertisements and marketing materials focused on the recruitment of racial and ethnic minority youth and LGBTQ+ youth, given the study’s interest in examining COVID-19 related health behaviors among minoritized youth. Interested individuals completed an online screener, and eligible participants who provided informed consent were invited to complete the survey.

For participants under 18 years old, we determined their capacity to consent using a brief, online assessment. Informed consent was then obtained electronically. Participants who completed the baseline survey received a digital $30 VISA card. Study procedures were approved by Northwestern University’s Institutional Review Board, and a waiver of parental permission was obtained for minor adolescent participants.

#### Analytic Sample

In total, 2395 eligible individuals provided informed consent. After data cleaning procedures were implemented to exclude potentially unreliable respondents and individuals who did not complete the survey, a final sample of 1055 remained. For this study, missing values (*n* = 7), response options of “Not listed” (*n* = 9), “Prefer not to answer” or “Not sure about the question” (*n* = 13) to demographics, were excluded, resulting in a final analytic sample of 1026.

### 2.2. Measures

#### 2.2.1. Measurement Development Process

COVID-19-specific measures were iteratively developed by the study team based on the existing public health literature and community feedback, with constructs related to COVID-19 information, motivation, and behavior being assessed. This scale was developed by the research team by adapting the measures used by Fisher et al. [19] in the initial empirical testing of the IMB model [19], with key issues related to COVID-19 prevention and mitigation effecting YYA. Sources for the information tested in this measure included the Centers for Disease Control and Prevention (CDC), Johns Hopkins Medicine, and Mayo Clinic [4,33,34]. Items were developed in Fall 2021, at which time universal masking procedures were still in place and the vaccine roll out had begun. The full measures and response options for each scale are available in the Appendices A-G.

#### 2.2.2. Information

Knowledge of COVID-19 prevention was assessed with 9 true/false items (Appendix A). Due to the evolving nature of the pandemic, three items which reflected CDC recommendations at the time of scale construction were no longer true when the survey closed (i.e., “I won’t need to wear a mask after I get vaccinated for COVID-19”, and “It is okay to stop social distancing, as long as you are wearing a mask and washing your hands frequently”). These items were dropped for final analyses.

#### 2.2.3. Motivation

Motivation was assessed using three separate constructs: attitudes, prevention stigma, and norms, which were developed by adapting existing measures used in HIV pre-exposure prophylaxis (PrEP) research [35]. These constructs were selected in accordance with Fisher’s explanation that motivation consists of personal motivation measuring beliefs/attitudes and social motivation measuring stigma and descriptive norms [36].

##### COVID-19 Beliefs/Attitudes

Beliefs and attitudes toward COVID-19 were assessed with 10 items with response options: Yes, No, or Unsure (Appendix B). Select, relevant common data element items from the CDC COVID-19 Community Survey Question Bank were used to assess beliefs/attitudes towards COVID-19 [37], with additional items devised by the study team as needed.

##### COVID-19 Prevention Stigma

Prevention stigma was assessed with 5 items (Appendix C). Responses were on a 5-point scale from strongly disagree to strongly agree, with higher scores indicating more prevention stigma associated with COVID-19. Given that validated scales specifically about COVID-19 were not available at the time of creating the survey, items assessing prevention stigma were modeled after existing measures used in the field of HIV prevention [36].

##### COVID-19 Norms

Norms were assessed with 4 items (Appendix D). Responses were on a 5-point scale from strongly disagree to strongly agree, with higher scores indicating more positive norms associated with COVID-19 prevention. Similar to prevention stigma, items were adapted from scales from the field of HIV prevention due to a lack of available metrics for COVID-19 early in the pandemic [36].

#### 2.2.4. Behavior

Items assessing behaviors for COVID-19 prevention including COVID-19 self-efficacy and COVID-19 behavioral intentions were developed by adapting items that measured behavior related to HIV prevention, specifically self-efficacy and intentions [36]. While the IMB model suggests measuring both objective abilities and self-efficacy [21], the cross-sectional nature of the survey and the wide variability in COVID-19 prevention resources made measuring actual behavior difficult. As such, measurements of behavioral intentions were used instead of self-reported behavior.

##### COVID-19 Self-efficacy

Participants reported how easy or difficult it would be to engage in 12 different behaviors related to responses to the COVID-19 pandemic (Appendix E). Responses were on a 4-point scale from very hard to do to very easy to do.

##### COVID-19 Prevention Intentions

Participants were assessed with 8 items on behavioral intentions regarding COVID-19 (Appendix F). Responses were on a 4-point scale from definitely will not do to definitely will do.

#### 2.2.5. Demographics

A detailed description of demographic measures is presented in the appendix (Appendix G). We included standard measures of age, race/ethnicity, sexual identity, gender identity, and gender modality.

### 2.3. Data Analysis

Data cleaning, recoding, and statistical analyses were conducted in RStudio version 4.2.1 [38]. Exploratory factor analysis (EFA) was conducted for each of the potential constructs listed above. Scree plots were generated for all EFAs to determine the optimal number of factors within the construct. Maximum likelihood estimation and oblimin rotation was used to identify variables that loaded into each factor. All preliminary results were reviewed, and poor loading and cross-loading variables were removed in a stepwise manner. Once a final factor structure was determined, model fit statistics were rerun to ensure a good fit, and variables within the factor were reviewed for consistent and coherent topics. Finally, factor scores using regression were computed for each participant and used in subsequent analyses, with the finalized models for each measure. The standard Cronbach’s alpha and mean scores for each factor were calculated. For subsequent analyses, we conducted bivariate linear regressions for assessing the association between demographic characteristics (age, race/ethnicity, sexual identity, gender and gender modality) and each factor score (*n* = 9).

## 3. Results

### 3.1. Demographics

Most participants were aged 18–21 (48.25%) and identified as Latinx (33.33%) or white (22.51%; Table 1). Nearly one-third of the sample identified as bisexual or pansexual (34.31%), 22.12% identified as gay or lesbian, and 21.93% identified as straight. Substantially fewer individuals identified as asexual/ace spectrum, queer, or questioning. The majority of the sample identified as cisgender (64.23%) and 32.26% identified as transgender and gender diverse. The sample primarily identified their gender as woman/girl (50.00%), man/boy (26.90%), or non-binary (16.37%).

### 3.2. Information, Motivation, and Behavioral Skills

#### 3.2.1. Information

An EFA was conducted on the 6 remaining COVID-19 information items (Appendix A). Parallel analysis and scree plot examination identified no ideal factor structure. Testing a single factor solution did not result in a good fitting model—root mean square error of approximation (RMSEA) = 0.06, 90% confidence intervals (CIs) [0.041, 0.078]. Therefore, subsequent analyses focus on the individual information questions. Overwhelmingly, participants provided the correct response to all 6 questions on the knowledge of COVID-19 prevention (Table 2). More than one-third of participants (36%) incorrectly said that the following statement was false: “In most places, people under 18 will need parental consent to get a COVID-19 test”.

#### 3.2.2. Motivation

##### Beliefs

An EFA on ten questions related to COVID-19 beliefs (Appendix B) identified that a two-factor solution was optimal. Parallel analysis and scree plot examination suggested a two-factor solution with 10 items, which was tested based on the theory. However, three items (D, F and I) which were cross-loaded were eliminated to improve model fit. In the final two-factor solution with seven items, the simple structure with each item loading on one and only one factor was achieved. The RMSEA indicated an acceptable fit at 0.05, 90% CIs [0.036, 0.074]. Factor 1 comprised two items that measured protection beliefs (α = 0.57), whereas Factor 2 comprised five items that measured prevention efficacy beliefs (α = 0.48).

##### Prevention Stigma

An EFA on five questions related to COVID-19 prevention stigma (Appendix C) identified that a one-factor solution was optimal. Parallel analysis and scree plot examination suggested a two-factor solution with five items, which was tested based on theory. However, item E was eliminated due to low loading. In the final one-factor solution with four items, the RMSEA indicated marginal fit at 0.1, 90% CIs [0.066, 0.139]. The sole factor for COVID-19 prevention stigma consisted of four items (α = 0.68).

##### Norms

An EFA on four questions related to motivation norms (Appendix D) identified that a one-factor solution was optimal. Parallel analysis and scree plot examination suggested a two-factor solution with four items; however, two items (B and C) were eliminated due to cross-loading and low factor loading in the test. This resulted in a one-factor solution comprised of two items (α = 0.51).

#### 3.2.3. Behavior

##### COVID-19 Self-efficacy

An EFA on 12 initial items related to individuals’ behaviors in response to the pandemic (Appendix E) identified that a three-factor solution was optimal. A parallel analysis and scree plot examination suggested a four-factor solution with 12 items, which was tested based on theory. However, four items (A, B, E and H) were eliminated due to low factor loadings. In the final three-factor solution with eight items, a simple structure with each factor highly loading on one factor and near-zero loadings on others was achieved. The RMSEA indicated an acceptable fit at 0.06, 90% CIs [0.042, 0.082]. The reliability of 3 factors was 0.74, 0.62 and 0.49, respectively. Factor 1—Protect Others was comprised of three items that assessed individuals’ behaviors protecting other such as encouraging others to wear masks or to social distance. Factor 2—COVID-19 Resources was comprised of 3 items that measured resource and help seeking such as calling the state’s helpline or seeking help for mental health if needed. Factor 3—Follow Prevention Instruction was comprised of two items that measured an individual’s prevention behavior with items such as wearing masks.

##### COVID-19 Prevention Intention

An EFA on eight initial items related to behavior intention in response to the pandemic (Appendix F) identified that a two-factor solution was optimal. Parallel analysis and scree plot examination suggested a three-factor solution with eight items, which was tested based on theory. However, two items (C, G) were eliminated due to low factor loadings. In the final two-factor solution with six items, the simple structure with each factor highly loading on one factor and near-zero loadings on others was achieved. The RMSEA indicated a good fit at 0.04, 90% CIs [0.012, 0.07]. The reliability of two factors were 0.77 and 0.73, respectively. Factor 1—Intentions to Social Distance included four items that assessed behavioral intentions to practice social distancing such as avoiding going out to an indoor restaurant, bar, or a family gathering. Factor 2—Testing intentions included two items that measured an individual’s intent on testing, including encouraging others or getting COVID-19 testing if they were having symptoms.

### 3.3. Bivariate Associations

#### 3.3.1. Information

Most significant associations between demographics and individual Information questions were for race/ethnicity (Table 2). White participants were the least likely to provide the correct answer to “In most places, people under 18 will need parental consent to get a COVID-19 test. (TRUE)”. AI/AN participants were the least likely to provide the correct answer to three items: “Antibiotics are effective at preventing COVID-19 infection (FALSE)”, “Only people over age 65 who get COVID-19 will require hospitalization (FALSE)”, and “There is no reason to get tested for COVID-19 if you do not have symptoms (FALSE)”. Although the proportion of correct responses was very high for the remaining two items, Black individuals had the lowest proportion of correct responses. Nearly all other demographic associations were not significant. The only exception was for sexual identity—straight participants were the least likely and queer participants were the most likely to provide the correct response for the item “Antibiotics are effective at preventing COVID-19 infection (FALSE)”.

#### 3.3.2. Motivation

##### Protection Beliefs

Asexual/ace spectrum individuals had significantly higher protection belief scores than straight individuals (unstandardized Beta (B) = 0.25; *p* = 0.03; Table 3). Individuals who identified as questioning their gender identity had higher protection belief scores than women/girls (B = 0.41; *p* < 0.01). Individuals who identified as being unsure of their gender modality had significantly higher protection belief scores than cisgender individuals (B = 0.42; *p* < 0.01).

##### Prevention Efficacy Beliefs

Individuals with a Latinx (B = −0.13; *p* = 0.03) or multiracial (B = −0.24; *p* = 0.01) race/ethnicity had significantly lower prevention efficacy belief scores than white participants. Individuals who identified as questioning their sexual identity had significantly higher scores than straight individuals (B = 0.36; *p* = 0.03), and individuals who identified as genderqueer had significantly higher scores than women/girls (B = 0.34; *p* = 0.02).

##### COVID-19 Prevention Stigma

Individuals who identified as AI/AN (B = 0.25; *p* = 0.01) or Black (B = 0.18; *p* = 0.03) had significantly higher prevention stigma scores than white individuals. Participants who identified as bisexual/pansexual (B = −0.18; *p* = 0.01), gay/lesbian (B = −0.18; *p* = 0.02), or queer (B = −0.26; *p* < 0.01) had significantly lower scores than straight participants. Nonbinary participants (B = −0.20; *p* = 0.01) had significantly lower scores than women/girls, and transgender and gender diverse participants (B = −0.21; *p* < 0.01) had significantly lower scores than cisgender participants.

##### Motivation Norms

Participants aged 18–21 (B = 0.38; *p* < 0.01) and 22–24 (B = 0.36; *p* < 0.01) had significantly higher motivation norms scores than those aged 14–17. Black (B = −0.16; *p* = 0.02) and Latinx (B = −0.12; *p* = 0.04) participants had significantly lower scores than white individuals. Participants who were questioning their sexual identity (B = −0.52; *p* < 0.01) had significantly lower scores than straight individuals.

#### 3.3.3. Behavior

##### Protect Others

Participants who identified as gay/lesbian (B = 0.24; *p* = 0.01) or queer (B = 0.20; *p* = 0.04) had significantly higher scores for engaging in behaviors to protect others than straight participants. No other demographic associations were significant (Table 4).

##### COVID-19 Resources

AI/AN (B = 0.35; *p* < 0.01) and Black (B = 0.30; *p* < 0.01) participants had significantly higher scores for accessing COVID-19 resources than white participants. However, participants who identified as asexual/ace spectrum (B = −0.37; *p* < 0.01) or bisexual/pansexual (B = −0.16; *p* = 0.03) had significantly lower scores than straight participants.

##### Follow Prevention Instructions

Participants aged 22–24 (B = 0.16; *p* = 0.03) had significantly higher scores for following prevention instructions than those aged 14–17 (Table 4). AI/AN (B = −0.20; *p* = 0.03), black (B = −0.25; *p* < 0.01), and multiracial (B = −0.28; *p* < 0.01) individuals had significantly lower scores than white participants. Queer individuals (B = 0.24; *p* < 0.01) had significantly higher scores than straight individuals. Individuals who identified as men/boys (B = −0.15; *p* = 0.01) had significantly lower scores than women/girls.

##### Intentions to Social Distance

Participants’ intentions to social distance significantly differed by race/ethnic identity, sexual identity, gender, and gender modality. AI/AN (B = −0.63; *p* < 0.01), black (B = −0.27; *p* < 0.01), Latinx (B = −0.40; *p* < 0.01), and multiracial (B = −0.55; *p* < 0.01) individuals had significantly lower scores for their intention to social distance than white individuals. Participants who identified as asexual/ace spectrum (B = 0.50; *p* < 0.01), bisexual/pansexual (B = 0.26; *p* < 0.01), gay/lesbian (B = 0.31; *p* < 0.01), and queer (B = 0.54; *p* < 0.01) had significantly higher scores than straight participants. Individuals who identified as nonbinary (B = 0.36; *p* < 0.01) had significantly higher scores than women/girls. Transgender and gender diverse individuals (B = 0.35; *p* < 0.01) and participants who reported being unsure if they were transgender (B = 0.27; *p* < 0.01) had significantly higher scores than cisgender individuals.

##### Testing Intentions

Participants aged 18–21 (B = 0.25; *p* < 0.01) and 22–24 (B = 0.26; *p* < 0.01) had significantly higher scores for intentions to test for COVID-19 than those aged 14–17. AI/AN (B = −0.34; *p* < 0.01), black (B = −0.31; *p* < 0.01), and multiracial (B = −0.29; *p* = 0.01), had significantly lower scores than white individuals. Bisexual/pansexual (B = 0.24; *p* < 0.01), gay/lesbian (B = 0.17; *p* = 0.03), and queer (B = 0.34; *p* < 0.01) individuals had significantly higher scores than straight individuals. Transgender and gender diverse (B = 0.14; *p* = 0.02) participants had significantly higher scores than cisgender participants.

## 4. Discussion

This study aimed to develop a measure to assess COVID-19 information, motivation, and behavioral skills, and explore the role of IMB factors in COVID-19 mitigation strategies for a diverse population of YYA ages 14–24. We iteratively developed a measure of COVID-19 risk and prevention behaviors through the adaptation of measures developed by Fisher et al. [19] in the initial empirical testing of the IMB model [19]. In our measure, we included include key issues related to COVID-19 prevention and mitigation affecting YYA. Our exploratory analysis demonstrated that the IMB model was useful for understanding how YYA engaged in COVID-19 prevention behaviors, and highlighting which populations may require additional intervention/support. Specifically, existing IMB scales were able to be adapted and the composite variables loaded into appropriate factors, except for one category. As such, IMB may be a good fit for modelling future COVID-19 prevention interventions and attempts to understand prevention behaviors among YYA. This echoes the previous research that highlighted the adaptability of the IMB model [18,25,35]. Here, we discuss the IMB factor analyses and then IMB factor score differences by sociodemographic characteristics.

### 4.1. IMB Factors

Although IMB models for other conditions and populations are commonly able to develop an information score from survey questions [21], we were unable to do so in this instance. The reasons for this may be two-fold: first, what could be considered as factually accurate information changed drastically during the data collection period and by the region in which the YYA was living (due to a rapidly changing prevention landscape; Hallas L, 2021). For example, in California, masks were mandated until 15 February 2022, whereas in Missouri, mandates ended on 30 September 2020 [39]. Second, opposing directives from local and state health departments led to different understandings of COVID-19 information. For most other diseases, this same variability is uncommon [40] in that uniform national recommendations are available. The highly political nature of COVID-19 information may have also impacted our ability to create a single information factor. COVID-19 has been characterized as an “infodemic”, leading to polarized opinions, widespread misinformation, and opposition to public health measures [41]. Political or social beliefs may influence one’s belief in misinformation about COVID-19, leading to low levels of information [30]; for other diseases, low information may be influenced primarily by a lack of education on a specific topic [35]. Further research using the IMB model for COVID-19 should investigate how knowledge may be affected by regional, temporal, and political variations among YYA, and develop contextually flexible measures of information, accordingly.

COVID-19 motivation was separated into four subscales: prevention beliefs, prevention efficacy beliefs, COVID-19 prevention stigma, and norms, similar to prior applications of the IMB model. The emergence of these subscales, in particular, the separation of beliefs into protection beliefs and prevention efficacy beliefs, may suggest that, in this population, the mechanisms behind desiring to protect oneself or others are different than the mechanisms which actually drive action, such as beliefs in getting tested, washing hands, and practicing social distancing. Further research is needed to fully understand how beliefs function within the COVID-19 context.

The COVID-19 behavior construct was designed with two subscales, self-efficacy and prevention intention. However, these subscales were further broken down to fully reflect the variance in COVID-19 prevention behaviors. Three factors emerged for self-efficacy: protecting others, ability to access COVID-19 resources, and ability to follow prevention instructions. This may indicate that the barriers and facilitators to successful prevention depend on who is being protected and who is providing the prevention supports. For example, YYA may find it easy to follow instructions, but have more difficulty accessing resources, as these are more dependent on community and familial supports. The prevention intention scale was broken down into two factors that related to intentions around specific prevention behaviors—testing and social distancing. This may indicate that different COVID-19 behaviors have different motivations and behavioral intentions associated with them, and therefore may require different intervention/messaging strategies.

### 4.2. Differences by Sexual Identity

Given the lack of information regarding COVID-19 prevention behaviors in sexual minority populations, a number of interesting patterns emerged when looking at IMB factors by sexual identity. COVID-19 prevention stigma was lower in bisexual/pansexual, gay/lesbian, and queer populations compared to straight respondents. Prior research has suggested that this may be related to existing practices around regular HIV testing and prevention that have been more normalized within the SGM community [42], which enabled easier stigma discussions in regards to COVID-19. Sexual minority respondents reported higher prevention behaviors across several constructs; this includes protecting others (gay/lesbian, queer), following prevention instructions (queer), intentions to social distance (asexual/ace spectrum, bisexual/pansexual, gay/lesbian, and queer), and testing intentions (bisexual/pansexual, gay/lesbian, and queer).

Because many SGM respondents were recruited from community centers, results may also reflect higher levels of social connectedness, which has been associated with greater prevention behaviors [43,44]. Of note, the exception to this pattern of higher prevention was for COVID-19 resources, including seeking help from doctors or for mental health needs, for which asexual/ace spectrum and bisexual/pansexual respondents had lower factor scores than straight participants. This may be related to structural healthcare factors that disproportionately impact bisexual/pansexual populations in particular [45], which resulted in poorer access to testing and prevention resources.

### 4.3. Differences by Gender Identity and Modality

Genderqueer respondents had significantly higher prevention efficacy beliefs than women/girls, whereas non-binary participants had significantly lower COVID-19 prevention stigma than women/girls. By gender modality, differences only existed for COVID-19 stigma, where transgender participants had significantly lower scores than cisgender participants. As in the case of sexual minority youth, the transgender community may be better prepared to manage norms and conversations around COVID-19 due to existing practices around HIV [46].

Men/boys reported significantly lower scores of the following prevention instructions. This aligns with prior research which suggests that girls/women display greater risk aversion, and therefore may be more likely to follow prevention instructions compared to boys/men [47]. Non-binary respondents (compared to women/girls) and transgender and gender diverse individuals (compared to cisgender participants) had significantly higher intentions to social distance. While this may seem contradictory to expected findings given known medical mistrust within gender minority populations [48], prior studies found that youth engaged in social distancing to improve their mental and social health [49]. Given that transgender and gender diverse youth experienced more mental health problems than cisgender youth during the pandemic [50], they may be more likely to engage in social distancing to protect themselves from further harm [49].

### 4.4. Differences by Race/Ethnicity

Significant differences by race/ethnicity were observed for motivation constructs. Prevention efficacy beliefs were significantly lower in Latinx and multiracial populations, COVID-19 stigma was significantly higher in AI/AN and black participants, and black and Latinx participants reported lower motivation norm scores than white individuals. These disparities could reflect medical mistrust among minoritized populations in regards to certain prevention/mitigation procedures [51]. The significant disparities in COVID-19 morbidity and mortality in black, Latinx, and AI/AN populations may have also contributed to a lower belief in the efficacy of prevention behaviors or lower motivations to engage in prevention [51,52].

Similarly, differences were observed between white and racial/ethnic minority YYA for behavioral factors. For example, AI/AN, black, and multiracial participants were significantly less likely to report intent to follow prevention instructions and to test for COVID-19 than white YYA. This may reflect the structural barriers which affected COVID-19 prevention adherence in many communities, including high rates of essential workers or difficulty social distancing [53]. AI/AN and black participants had higher scores on the intention to use COVID-19 resources scores than white participants. On the surface, this is contradictory to prior research, which indicated a dearth in COVID-19 resources in many racial/ethnic minority communities [54]; however, the measure only reflects intentions, not whether participants were actually able to access these resources. While this may indicate success in national initiatives to increase resources in underserved communities, it also may suggest that our sample was not necessarily representative of populations at the highest level of material need throughout the COVID-19 pandemic. Further longitudinal research is needed to fully understand this mechanism [55].

### 4.5. Differences by Age

Older participants reported higher prevention behaviors across several factors. For example, participants ages 18–21 and 22–24 reported higher motivation norms than those ages 14–17. The stage of adolescence has been characterized by a sense of invulnerability to risk and self-focus [56], which may have led to a lower likelihood to engage in prevention behaviors. Older participants scored higher on testing intentions and on following prevention. While this may be related to the stage of development, this also could be linked to the significant number of testing barriers which existed for those under the age of 18, including needing parental consent and needing assistance with transit [57]. This may point to the need for additional support for younger populations to help bolster prevention behaviors moving forward, including potential policy changes to allow younger individuals to independently consent to COVID-19-related medical services [58].

### 4.6. Limitations

While this study contributes to the literature on YYA’s COVID-19 prevention strategies, it is important to consider several limitations that may affect the generalizability and interpretation of the findings. First, this study relied on self-reported data, which may introduce bias and inaccuracies into the results. For example, participants may have underreported or overreported their experiences due to social desirability bias or memory recall issues. Second, data collection occurred online, which may have resulted in the sampling bias towards individuals with a greater access to technology and the Internet. Third, due to diversity limitations in our sample, we were unable to examine the influence of being both a racial/ethnic and sexual minority, which may have influenced on YYA’s response. YYA’s who identify as both a racial/ethnic and sexual minority may experience a unique confluence of risk and protective factors that are not well represented in decomposition studies such as ours. Future research on these potential associations is warranted. Lastly, recruitment into the study relied on a convenience sample; this may have led to a biased sample of participants who are more connected to social media and more likely to engage in online surveys. Future research using a more diverse sample and multiple methods of data collection may help to overcome some of these limitations and provide a more comprehensive understanding of YYA’s response to pandemics.

## 5. Conclusions

YYA, especially SGM and racial/ethnic minority YYA, were disproportionately impacted by the COVID-19 pandemic. Understanding how YYA make decisions related to the pandemic is critical to inform responses to future pandemics and ensure equitable access to preventive resources for minoritized populations. In our study of YYA, we found that the IMB model could be adapted for use in a COVID-19 context, and that all constructs except information could be combined into coherent factors. Differences in knowledge, motivation, and behavioral intentions were observed across all demographic factors, with some striking differences among sexual minorities and racial/ethnic minorities. Future studies should be dedicated to examining how the embodiment of multiple marginalized identities (i.e., identifying as both a sexual and racial/ethnic minority) may influence future pandemic prevention behaviors. Despite these limitations, results from this study can be used to ensure that future messaging and resources are tailored for populations that already experience medical mistrust and are disengaged from the healthcare system, particularly among sexual minorities and racial/ethnic minorities.

## Figures and Tables

**Table 1.** Study Participant Demographics (*N* = 1026).

	Demographics	*N* (%)
Age, Years		
	14–17	172 (16.76)
	18–21	495 (48.25)
	22–24	359 (34.99)
Race/Ethnicity		
	White	231 (22.51)
	American Indian or Alaska Native	97 (9.45)
	Asian	75 (7.31)
	Black	181 (17.64)
	Latinx	342 (33.33)
	Multiracial	100 (9.75)
Sexual Identity		
	Straight	225 (21.93)
	Asexual/Ace Spectrum	53 (5.17)
	Bisexual/Pansexual	352 (34.31)
	Gay/Lesbian	227 (22.12)
	Queer	147 (14.33)
	Questioning	22 (2.14)
Gender		
	Woman/Girl	513 (50.00)
	Agender	10 (0.97)
	Gender Queer	29 (2.83)
	Man/Boy	276 (26.90)
	Non-binary	168 (16.37)
	Questioning	30 (2.92)
Gender Modality		
	Cisgender	659 (64.23)
	Trans and Gender Diverse	331 (32.26)
	Not sure	36 (3.51)
	Total	1026

**Table 2.** Demographic Associations with COVID-19 Prevention Knowledge (Information).

	Knowledge of COVID-19 Prevention											
	Antibiotics Are Effective at Preventing COVID-19 Infection. (FALSE)		Only People over Age 65 Who Get COVID-19 Will Require Hospitalization. (FALSE)		In Most Places, People under 18 Will Need Parental Consent to Obtain a COVID-19 Test. (TRUE)		Once Infected with COVID-19, It Can Take 2 to 14 Days to Show Symptoms. (TRUE)		A Loss of Smell or Taste Is a Symptom of COVID-19. (TRUE)		There Is No Reason to Get Tested for COVID-19 If You do Not Have Symptoms. (FALSE)	
Demographics	N (%)	*p*-Value	N (%)	*p*-Value	N (%)	*p*-Value	N (%)	*p*-Value	N (%)	*p*-Value	N (%)	*p*-Value
Age, Years		0.14		0.14		0.51		0.25		0.98		0.84
14–17	132 (76.74)		158 (91.86)		117 (68.02)		168 (97.67)		170 (98.84)		161 (93.60)	
18–21	388 (78.38)		451 (91.11)		314 (63.43)		486 (98.18)		490 (98.99)		469 (94.75)	
22–24	298 (83.01)		340 (94.71)		227 (63.23)		346 (96.38)		355 (98.89)		340 (94.71)	
Race/Ethnicity		**<0.01**		**<0.01**		**0.03**		**0.01**		***p* < 0.01**		***p* < 0.001**
White	**199 (86.15)**		**218 (94.37)**		**131 (56.71)**		**229 (99.13)**		**231 (100.00)**		**227 (98.27)**	
American Indian or Alaska Native	**66 (68.04)**		**83 (85.57)**		**72 (74.23)**		**92 (94.85)**		**97 (100.00)**		**82 (84.54)**	
Asian	**55 (73.33)**		**70 (93.33)**		**46 (61.33)**		**75 (100.00)**		**75 (100.00)**		**74 (98.67)**	
Black	**137 (75.69)**		**160 (88.40)**		**114 (62.98)**		**171 (94.48)**		**174 (96.13)**		**169 (93.37)**	
Latinx	**275 (80.41)**		**321 (93.86)**		**233 (68.13)**		**334 (97.66)**		**339 (99.12)**		**322 (94.15)**	
Multiracial	**86 (86.00)**		**97 (97.00)**		**62 (62.00)**		**99 (99.00)**		**99 (99.00)**		**96 (96.00)**	
Sexual Identity		**<0.01**		0.11		0.48		0.58		0.41		0.25
Straight	**156 (69.33)**		208 (92.44)		151 (67.11)		220 (97.78)		221 (98.22)		210 (93.33)	
Asexual/Ace Spectrum	**45 (84.91)**		47 (88.68)		30 (56.60)		51 (96.23)		52 (98.11)		50 (94.34)	
Bisexual/Pansexual	**289 (82.10)**		327 (92.90)		230 (65.34)		346 (98.30)		350 (99.43)		333 (94.60)	
Gay/Lesbian	**184 (81.06)**		207 (91.19)		147 (64.76)		218 (96.04)		223 (98.24)		212 (93.39)	
Queer	**127 (86.39)**		142 (96.60)		86 (58.50)		144 (97.96)		147 (100.00)		145 (98.64)	
Questioning	**17 (77.27)**		18 (81.82)		14 (63.64)		21 (95.45)		22 (100.00)		20 (90.91)	
Gender		0.53		0.15		0.12		0.13		0.52		0.08
Woman/Girl	405 (78.95)		476 (92.79)		347 (67.64)		500 (97.47)		509 (99.22)		**482 (93.96)**	
Agender	10 (100.00)		10 (100.00)		4 (40.00)		10 (100.00)		10 (100.00)		**10 (100.00)**	
Gender Queer	24 (82.76)		28 (96.55)		18 (62.07)		26 (89.66)		28 (96.55)		**25 (86.21)**	
Man/Boy	217 (78.62)		247 (89.49)		172 (62.32)		269 (97.46)		271 (98.19)		**259 (93.84)**	
Non-binary	136 (80.95)		158 (94.05)		97 (57.74)		165 (98.21)		167 (99.40)		**165 (98.21)**	
Questioning	26 (86.67)		30 (100.00)		20 (66.67)		30 (100.00)		30 (100.00)		**29 (96.67)**	
Gender Modality		0.31		0.11		0.10		0.61		0.74		0.49
Cisgender	516 (78.30)		603 (91.50)		436 (66.16)		642 (97.42)		651 (98.79)		619 (93.93)	
Trans and Gender Diverse	272 (82.18)		310 (93.66)		197 (59.52)		322 (97.28)		328 (99.09)		317 (95.77)	
Not Sure	30 (83.33)		36 (100.00)		25 (69.44)		36 (100.00)		36 (100.00)		34 (94.44)	
Frequency of Responses	Correct	Incorrect	Correct	Incorrect	Correct	Incorrect	Correct	Incorrect	Correct	Incorrect	Correct	Incorrect
(N = 1026)	818 (0.80)	208 (0.20)	949 (0.92)	77 (0.08)	658 (0.64)	368 (0.36)	1000 (0.97)	26 (0.03)	1015 (0.99)	11 (0.01)	970 (0.95)	56 (0.05)

Note. significant results are bolded.

**Table 3.** Bivariate Associations Between Demographics and Factor Scores for COVID-19 Beliefs, Stigma, and Norms (Motivation).

	Belief Factor 1 (Protection)				Belief Factor 2 (Prevention Efficacy Beliefs)				Stigma Factor (COVID-19 Stigma)				Norms Factor (Motivation Norms)			
Demographics	Coefficient	Std. Error	t	*p*	Coefficient	Std. Error	t	*p*	Coefficient	Std. Error	t	*p*	Coefficient	Std. Error	t	*p*
Age, Years																
14–17	Ref				Ref				Ref				Ref			
18–21	0.10	0.07	1.46	0.15	0.06	0.07	0.92	0.36	−0.07	0.07	−1.01	0.31	**0.38**	**0.06**	**6.03**	**<0.01**
22–24	0.05	0.07	0.69	0.49	0.03	0.07	0.37	0.71	0.01	0.08	0.07	0.95	**0.36**	**0.07**	**5.49**	**<0.01**
Race/Ethnicity																
White	Ref				Ref				Ref				Ref			
American Indian or Alaska Native	−0.01	0.09	−0.08	0.93	−0.04	0.09	−0.42	0.68	**0.25**	**0.10**	**2.47**	**0.01**	−0.14	0.09	−1.57	0.12
Asian	0.00	0.10	0.05	0.96	−0.10	0.10	−0.98	0.33	−0.04	0.11	−0.32	0.75	−0.13	0.10	−1.40	0.16
Black	−0.10	0.08	−1.37	0.17	−0.09	0.07	−1.30	0.19	**0.18**	**0.08**	**2.16**	**0.03**	**−0.16**	**0.07**	**−2.31**	**0.02**
Latinx	0.07	0.06	1.01	0.31	**−0.13**	**0.06**	**−2.12**	**0.03**	0.08	0.07	1.07	0.28	**−0.12**	**0.06**	**−2.02**	**0.04**
Multiracial	−0.08	0.09	−0.85	0.40	**−0.24**	**0.09**	**−2.78**	**0.01**	0.08	0.10	0.78	0.43	−0.13	0.09	−1.50	0.13
Sexual Identity																
Straight	Ref				Ref				Ref				Ref			
Asexual/Ace Spectrum	**0.25**	**0.12**	**2.19**	**0.03**	0.15	0.11	1.35	0.18	−0.23	0.13	−1.84	0.07	−0.06	0.11	−0.57	0.57
Bisexual/Pansexual	0.11	0.06	1.68	0.09	0.08	0.06	1.28	0.20	**−0.18**	**0.07**	**−2.52**	**0.01**	−0.01	0.06	−0.12	0.90
Gay/Lesbian	−0.03	0.07	−0.37	0.71	0.13	0.07	1.86	0.06	**−0.18**	**0.08**	**−2.32**	**0.02**	0.05	0.07	0.72	0.47
Queer	0.14	0.08	1.74	0.08	0.08	0.08	1.09	0.28	**−0.26**	**0.09**	**−3.00**	**<0.01**	0.07	0.08	0.86	0.39
Questioning	0.12	0.17	0.72	0.47	**0.36**	**0.16**	**2.20**	**0.03**	0.13	0.18	0.68	0.50	**−0.52**	**0.16**	**−3.26**	**<0.01**
Gender																
Woman/Girl	Ref				Ref				Ref				Ref			
Agender	−0.08	0.24	−0.35	0.73	0.12	0.23	0.52	0.61	−0.44	0.26	−1.68	0.09	0.02	0.23	0.09	0.93
Gender Queer	0.08	0.14	0.53	0.59	**0.34**	**0.14**	**2.43**	**0.02**	−0.03	0.16	−0.20	0.84	0.00	0.14	−0.01	0.99
Man/Boy	−0.07	0.06	−1.26	.21	−0.10	0.05	−1.82	0.07	0.05	0.06	0.73	0.46	0.00	0.05	0.02	0.98
Non-binary	0.08	0.07	1.16	.25	−0.06	0.07	−0.94	0.35	**−0.20**	**0.07**	**−2.67**	**0.01**	0.05	0.06	0.77	0.44
Questioning	**0.41**	**0.14**	**2.90**	**<0.01**	0.10	0.14	0.70	0.48	−0.15	0.16	−0.99	0.32	0.07	0.14	0.54	0.59
Gender Modality																
Cisgender	Ref				Ref				Ref				Ref			
Trans and Gender Diverse	**0.10**	**0.05**	**1.93**	**0.05**	0.00	0.05	−0.06	0.96	**−0.21**	**0.06**	**−3.74**	**<0.01**	0.06	0.05	1.33	0.18
Not Sure	**0.42**	**0.13**	**3.27**	**<0.01**	0.07	0.13	0.58	0.56	0.23	0.14	−1.59	0.11	0.05	0.12	0.39	0.70

Note. significant results are bolded.

**Table 4.** Bivariate Associations Between Demographics and Factor Scores for COVID-19 Self-efficacy and Prevention Intention (Behavior).

	Self-Efficacy Factor 1 (Protect Others)				Self-Efficacy Factor 2 (COVID-19 Resources)				Self-Efficacy Factor 3 (Follow Prevention Instruction)				Prevention Intention Factor 1 (Intentions to Social Distance)				Prevention Intention Factor 2 (Testing Intentions)			
Demographics	Coefficient	Std. error	t-Value	*p*-Value	Coefficient	Std. error	t-Value	*p*-Value	Coefficient	Std. error	t-Value	*p*-Value	Coefficient	Std. error	t-Value	*p*-Value	Coefficient	Std. error	t-Value	*p*-Value
Age, Years																				
14–17	Ref				Ref				Ref				Ref				Ref			
18–21	−0.04	0.08	−0.46	0.64	−0.04	0.07	−0.48	0.63	0.13	0.07	1.90	0.06	−0.01	0.08	−0.13	0.89	**0.25**	**0.08**	**3.21**	**<0.01**
22–24	−0.11	0.08	−1.31	0.19	−0.04	0.08	−0.57	0.57	**0.16**	**0.07**	**2.22**	**0.03**	0.05	0.08	0.66	0.51	**0.26**	**0.08**	**3.16**	**<0.01**
Race/Ethnicity																				
White	Ref				Ref				Ref				Ref				Ref			
American Indian or Alaska Native	−0.02	0.11	−0.18	0.86	**0.35**	**0.10**	**3.47**	**<0.01**	**−0.20**	**0.09**	**−2.16**	**0.03**	**−0.63**	**0.10**	**−6.05**	**<0.01**	**−0.34**	**0.11**	**−3.22**	**<0.01**
Asian	0.14	0.12	1.17	0.24	0.18	0.11	1.62	0.10	**−0.20**	**0.10**	**−1.94**	**0.05**	0.15	0.12	1.34	0.18	0.02	0.12	0.21	0.83
Black	0.17	0.09	1.85	0.06	**0.30**	**0.08**	**3.64**	**<0.01**	**−0.25**	**0.08**	**−3.36**	**<0.01**	**−0.27**	**0.09**	**−3.12**	**<0.01**	**−0.31**	**0.09**	**−3.59**	**<0.01**
Hispanic	−0.01	0.08	−0.12	0.90	0.07	0.07	0.95	0.34	−0.08	0.07	−1.30	0.19	**−0.40**	**0.07**	**−5.47**	**<0.01**	−0.09	0.07	−1.21	0.23
Multiracial	−0.19	0.11	−1.78	0.08	0.09	0.10	0.87	0.38	**−0.28**	**0.09**	**−3.11**	**<0.01**	**−0.55**	**0.10**	**−5.28**	**<0.01**	**−0.29**	**0.10**	**−2.81**	**0.01**
Sexual Identity																				
Straight	Ref				Ref				Ref				Ref				Ref			
Asexual/Ace Spectrum	−0.02	0.14	−0.12	0.91	**−0.37**	**0.13**	**−2.92**	**<0.01**	0.13	0.12	1.09	0.28	**0.50**	**0.13**	**3.75**	**<0.01**	0.08	0.13	0.63	0.53
Bi/Pan	0.07	0.08	0.94	0.35	**−0.16**	**0.07**	**−2.24**	**0.03**	0.09	0.07	1.42	0.16	**0.26**	**0.08**	**3.40**	**<0.01**	**0.24**	**0.08**	**3.16**	**<0.01**
Gay/Les	**0.24**	**0.09**	**2.77**	**0.01**	0.01	0.08	0.19	0.85	0.04	0.07	.50	0.62	**0.31**	**0.08**	**3.71**	**<0.01**	0.17	0.08	2.11	0.03
Queer	**0.2**0	**0.10**	**2.10**	**0.04**	−0.04	0.09	−0.45	0.65	**0.24**	**0.08**	**2.92**	**<0.01**	**0.54**	**0.09**	**5.82**	**<0.01**	**0.34**	**0.09**	**3.64**	**<0.01**
Questioning	0.09	0.20	0.43	0.67	−0.22	0.18	−1.22	0.22	−0.13	0.17	−0.74	0.46	0.01	0.20	0.07	0.95	−0.05	0.20	−0.24	0.81
Gender																				
Woman/Girl	Ref				Ref				Ref				Ref				Ref			
Agender	0.30	0.29	1.03	0.30	0.25	0.26	0.93	0.35	0.19	0.24	.78	0.43	0.16	0.28	0.57	0.57	−0.08	0.28	−0.29	0.77
Gender Queer	0.02	0.17	0.13	0.90	−0.06	0.16	−0.36	0.72	−0.18	0.15	−1.26	0.21	0.25	0.17	1.47	0.14	0.17	0.17	0.98	0.33
Man/Boy	−0.03	0.07	−0.39	0.70	0.07	0.06	1.20	0.23	**−0.15**	**0.06**	**−2.63**	**0.01**	−0.04	0.07	−0.67	0.50	−0.10	0.07	−1.51	0.13
Non-binary	0.13	0.08	1.63	0.10	−0.10	0.07	−1.29	0.20	0.07	0.07	1.00	0.32	**0.36**	**0.08**	**4.52**	**<0.01**	**0.16**	**0.08**	**1.98**	**0.05**
Questioning	−0.05	0.17	−0.32	0.75	−0.16	0.16	−1.00	0.32	0.07	0.14	.48	0.63	0.21	0.17	1.26	0.21	0.20	0.17	1.23	0.22
Gender Modality																				
Cisgender	Ref				Ref				Ref				Ref				Ref			
Trans and Gender Diverse	0.10	0.06	1.55	0.12	−0.09	0.06	−1.60	0.11	0.09	0.05	1.82	0.07	**0.35**	**0.06**	**5.93**	**<0.01**	**0.14**	**0.06**	**2.37**	**0.02**
Not Sure	0.04	0.16	0.23	0.82	−0.23	0.14	−1.60	0.11	0.17	0.13	1.26	0.21	**0.27**	**0.15**	**1.82**	**<0.01**	0.21	0.15	1.40	0.16

Note. significant results are bolded.

## Data Availability

The data presented in this study are available on request from the Northwestern University. The data are not publicly available due to privacy restrictions.

## Appendix A

**Table A1.** Final IMB Measure Items—Information.

Information: COVID-19 Knowledge		
Items		
Please indicate, to the best of your knowledge, whether the following statements are true or false.		
B.	Antibiotics are effective at preventing COVID-19 infection.	
C.	Only people over age 65 who get COVID-19 will require hospitalization.	
E.	In most places, people under 18 will need parental consent to get a COVID-19 test.	
G.	Once infected with the coronavirus, it can take 2 to 14 days to show symptoms.	
J.	A loss of smell or taste is a symptom of COVID-19.	
D.	There is no reason to get tested for COVID-19 if you do not have symptoms.	
A.	Healthy people should practice social distancing.	Eliminated
H.	I won’t need to wear a mask after I get vaccinated for COVID-19.	Eliminated
F.	It is okay to stop social distancing, as long as you are wearing a mask and washing your hands frequently.	Eliminated

## Appendix B

**Table A2.** Final IMB Measure Items—Motivation: COVID-19 Beliefs.

Motivation: COVID-19 Beliefs			
Items		Factor Loadings	
Below are a series of statements about COVID-19. Please indicate whether such statement is true for you by selecting yes, no, or unsure.		Factor 1 (Protection)	Factor 2 (Prevention Efficacy Beliefs)
A.	I believe I can protect myself from COVID-19.	0.40	
B.	I believe I can protect others from COVID-19.	0.99	
C.	I believe that COVID-19 is a serious disease.		0.31
E.	I believe that getting testing for COVID-19 if I am experiencing symptoms is important to stopping the spread of the virus.		0.37
G.	I believe that washing my hands regularly can prevent me and others around me from getting COVID-19.		0.29
H.	I believe that continuing to social distance is needed to prevent those around me from getting COVID-19.		0.52
j.	I believe it is everyone’s job in the community to work together to stop COVID-19.		0.52
D.	I believe that I am at risk of being infected with COVID-19.	Eliminated	
F.	I believe that wearing a mask will help stop the spread of the virus in my community.	Eliminated	
I.	I believe it is unlikely that I would get COVID-19 from my close friends or family.	Eliminated	

## Appendix C

**Table A3.** Final IMB Measure Items—Motivation: COVID-19 Stigma.

Motivation: COVID-19 Stigma		
Items		Factor Loadings
Using the scale provided, please rate how much you agree or disagree with the following statements by selecting strongly disagree, disagree, neutral, agree or strongly agree:		Stigma Factor (COVID-19 Stigma)
A.	It would be embarrassing to get a COVID-19 test.	0.67
B.	I wouldn’t want my friends knowing that I had COVID-19.	0.60
C.	Wearing a face mask in public is embarrassing.	0.53
D.	If I found out a friend who I had recently hung out with tested positive for COVID-19, I would be annoyed with them.	0.60
E.	If I got a COVID-19 test, I wouldn’t tell others around me, even if it came back negative.	Eliminated

## Appendix D

**Table A4.** Final IMB Measure Items—Motivation: COVID-19 Norms.

Motivation: COVID-19 Norms		
Items		Factor Loadings
Using the scale provided, please rate how much you agree or disagree with the following statements by selecting strongly disagree, disagree, neutral, agree or strongly agree:		Norms Factor (Motivation Norms)
A.	Most of my friends and family have abided by social distancing rules when possible.	0.60
D.	A lot of people I know have gotten tested for COVID-19.	0.60
B.	In my community, it is normal to wear a mask.	Eliminated
C.	I think my parents would be supportive if I wanted to get tested for COVID-19.	Eliminated

## Appendix E

**Table A5.** Final IMB Measure Items—Behavior: COVID-19 Self-Efficacy.

Behavior: COVID-19 Self-Efficacy				
Items		Factor Loadings		
How easy or difficult would the following be to do? Please select one option from the following options: very hard to do, somewhat hard, somewhat easy to do, and very easy to do.		Factor 1 (Protect Others)	Factor 2 (COVID-19 Resources)	Factor 3 (Follow Prevention Instruction)
C.	Wear a mask when I am hanging out with my friends.			0.46
D.	Wash my hands with soap and warm water for at least 20 s after going out or interacting with people.			0.76
F.	Call my state’s helpline or another source if I am having trouble finding COVID-19 testing.		0.45	
G.	Go to a doctor if I think I have COVID-19.		0.77	
I.	Seek out help if I thought my mental health was being impacted by the pandemic.		0.53	
J.	Encourage others around me to wear masks.	0.57		
K.	Say no to a friend if they ask me to hang out because I am social distancing.	0.58		
L.	Encourage friends to social distance.	0.91		
A.	Quarantine for 2 weeks if I think I may have been exposed to COVID-19.	Eliminated		
B.	Wear a mask when I am at the store or running errands.	Eliminated		
E.	Figure out where around me COVID-19 testing is available.	Eliminated		
H.	Tell my family or people I live with that I wanted to get tested for COVID-19.	Eliminated		

## Appendix F

**Table A6.** Final IMB Measure Items—Behavior: COVID-19 Prevention Intension.

Behavior: COVID-19 Prevention Intention			
Items		Factor Loadings	
How easy or difficult would the following be to do? Please select one option from the following options: definitely will not do, probably will not do, probably will do, and definitely will do.		Factor 1 (Intentions to Social Distance)	Factor 2 (Testing Intentions)
A.	Get a COVID-19 test if/when I have symptoms or am exposed		0.67
B.	Encourage others around me to get COVID-19 tests if they are having symptoms.		0.87
D.	Social distance from people not in my household.	0.54	
E.	Avoid going out to an indoor restaurant, bar, or club.	0.80	
F.	Avoid visiting with older (60+ years) family members.	0.60	
H.	Avoid going to a family gathering like a birthday party or wedding or funeral.	0.76	
C.	Wear a mask when I am in public.	Eliminated	
G.	Wash my hands with soap and warm water for at least 20 s after going out or interacting with people.	Eliminated	

## Appendix G

**Table A7.** Demographic Measures.

Measures	Descriptions
Age	Age was assessed by asking participants, “How old are you right now?” Participants were placed into one of three age group categories: (1) 14–17 years old, (2) 18–21 years old, and (3) 22–24 years old.
Race/Ethnicity	Two questions were used to assess race/ethnicity. Participants’ race was collected via the following question: “How do you describe your race? [Choose all that apply]”. Response options included (1) American Indian or Alaska Native, (2) Black or African American, (3) Asian, (4) Native Hawaiian or Other Pacific Islander, (5) White, (6) Not listed, and (7) Prefer not to answer. Latinx ethnicity was assessed with the question “Are you of Hispanic, Latinx, or Spanish origin?” Response options included (1) Yes, (2) No, and (3) Prefer not to answer. Individuals who responded “Not listed” to the Race question and provided write-in responses were recoded based on their responses. If participants wrote “Hispanic” or similar phrases, we assigned their response to the Latinx ethnicity question as “Yes”. If participants wrote a race that was listed in the race question, we assigned their race based on their write-in responses. Others remained as “Not listed”. Next, we combined the race and Latinx ethnicity responses. Participants who selected more than one option were categorized as 8) Multiracial. Participants who responded “Yes” to the Latinx ethnicity question were categorized as “Hispanic” regardless of how many options they selected for the race question. Additionally, we reviewed responses for individuals who responded “Prefer not to answer” to both questions and assigned their race/ethnicity based on their responses for race/ethnicity in the screener. Finally, race/ethnicity was collapsed into seven analytic groups: (1) American Indian/Alaska Native, (2) Asian, (3) Black or African American, (4) Hispanic, (5) Native Hawaiian or Other Pacific Islander, (6) Multiracial, and (7) Not listed.
Sexual Orientation	Sexual orientation was assessed by asking participants, “Which of the following best describes your sexual orientation at this time? [Choose all that apply]”. Response options included (1) Asexual or asexual spectrum, (2) Bisexual or pansexual, (3) Gay or lesbian, (4) Straight (heterosexual), (5) Queer, (6) Questioning my sexual orientation, (7) Not listed, and (8) Prefer not to respond. Individuals who responded “Not listed” were asked to provide a write-in response. For individuals who selected only one option for sexual orientation, we assigned their sexual orientation based on this selection. For individuals who selected more than one option, we then asked “If you could only pick one term to describe your sexual orientation, which would you pick?” and assigned their sexual orientation based on their specified selection among the first seven options listed above. Individuals who selected more than one option were also able to provide a write-in response. Finally, we assigned sexual orientation for participants who provided write-in responses (*n* = 10). For example, participants who wrote “Aromantic” were categorized as “Asexual or asexual spectrum” (*n* = 2) and participants who wrote “Omnisexual” were categorized as “Bisexual or pansexual” (*n* = 4).
Gender Identity	Gender identity was assessed by asking, “Which of the following terms best describes your gender at this time? [Choose all that apply]”. Response options included (1) Woman/Girl, (2) Man/Boy, 3) Two-spirit, (4) Non-binary, (5) Agender, (6) Genderqueer, (7) Questioning my gender identity, (8) Not listed, and (9) Prefer not to respond. Individuals who responded “Not listed” were asked to provide a write-in response, and were assigned gender identity based on their response (*n* = 18). For example, participants who wrote, “Gender Fluid” were categorized as “Non-binary”.
Gender Modality	First, participants were asked “Some people use the term transgender to describe themselves when their gender does not align with the sex they were assigned at birth. Do you identify as transgender?” Response options included (1) Yes, (2) No, (3) Prefer not to respond, (4) I’m not sure if I identify as transgender, and (5) I’m not sure what this question is asking. Using responses to this question and the Gender Identity question, we constructed Gender Modality including (1) Cisgender, (2) Trans and gender diverse, and (3) Not sure. Specifically, individuals who reported their Gender Identity as “man/boy” and “women/girl” and did not report a transgender gender modality were categorized as cisgender.
